# Anterior referencing of tibial slope in total knee arthroplasty considerably influences knee kinematics: a musculoskeletal simulation study

**DOI:** 10.1007/s00167-017-4561-3

**Published:** 2017-05-12

**Authors:** Marco A. Marra, Marta Strzelczak, Petra J. C. Heesterbeek, Sebastiaan A. W. van de Groes, Dennis W. Janssen, Bart F. J. M. Koopman, Ate B. Wymenga, Nico J. J. Verdonschot

**Affiliations:** 1 Orthopaedic Research Laboratory, Radboud Institute for Health Sciences, Radboud University Medical Center, P.O. Box 9101, 6500 HB Nijmegen, The Netherlands; 2 Sint Maartenskliniek Research, Postbus 9011, 6500 GM Nijmegen, The Netherlands; 3 Orthopaedic Department, Radboud University Medical Center, Postbus 9101, 6500 HB Nijmegen, The Netherlands; 4 Department of Biomechanical Engineering, University of Twente, Postbus 217, 7500 AE Enschede, The Netherlands; 5 Sint Maartenskliniek Orthopaedics, Postbus 9011, 6500 GM Nijmegen, The Netherlands

**Keywords:** Posterior, Tibial, Slope, Musculoskeletal, Computer, Model, Force, Dependent, Kinematics, Simulation, Knee, Arthroplasty, Biomechanics, CR, TKA

## Abstract

**Purpose:**

In total knee arthroplasty (TKA), the posterior tibial slope is not always reconstructed correctly, and the knee ligaments may become too tight in flexion. To release a tight flexion gap, surgeons can increase the posterior tibial slope using two surgical resection techniques: the anterior tibial cortex (ACR) or the centre of tibial plateau (CPR) referencing. It is not known how this choice affects the knee laxity and function during activities of daily living. The aim of this study was to investigate the effect of tibial slope on knee laxity, kinematics and forces during a squatting activity using computer simulation techniques. We hypothesised that the effects depend on the referencing technique utilised.

**Methods:**

A validated musculoskeletal model of TKA was used. Knee laxity tests were simulated in flexion and extension. Then, a squat motion was simulated to calculate: movement of the tibiofemoral joint (TFJ) contact points and patello-femoral joint (PFJ) contact force. All analyses were repeated with more anterior (−3°), neutral (0°), and more posterior tibial slope (+3°, +6°, +9°), and with two referencing techniques (ACR, CPR).

**Results:**

Knee laxities increased dramatically with more posterior slope with the ACR technique (up to 400%), both in flexion and in extension. The CPR technique, instead, had much smaller effects (up to 42% variations). During squatting, more slope with the ACR technique resulted in larger movements of the TFJ contact point. The PFJ contact force decreased considerably with more slope with the CPR technique (12% body weight reduction every 3° more posterior slope), thanks to the preservation of the patellar height and quadriceps–femur load sharing.

**Conclusion:**

ACR technique alters considerably the knee laxity, both in flexion and extensions, and surgeons should be cautious about its use. More slope with CPR technique induces more favourable TFJ kinematics and loading of the knee extensor apparatus and does not substantially alter knee laxity. Preferably, the tibial slope resection should be pre-planned thoroughly and performed using CPR technique as accurately as possible. Surgeons can directly translate the results of this study into the clinical practice.

**Electronic supplementary material:**

The online version of this article (doi:10.1007/s00167-017-4561-3) contains supplementary material, which is available to authorized users.

## Introduction

A successful total knee arthroplasty (TKA) should reduce knee pain and restore function to normal levels. For this purpose, the reconstructed tibiofemoral joint (TFJ) should be stable throughout the whole range of knee flexion. The posterior tibial slope was shown to affect substantially the knee laxity, hence the stability, in a cadaveric study [16].

In conventional TKA, the posterior tibial slope cannot always be reconstructed correctly, due to uncertainty of the surgical instruments and a large inter-patient variability (95% CI [1.0°, 15.8°]) [8]. Often, an arbitrary angle of posterior slope is aimed for (3° or 7° are common choices) according to the recommendation of the prosthesis manufacturer. In cruciate-retaining (CR)-TKA, particularly, an insufficient posterior tibial slope may result in flexion gap tightness and reduced post-operative flexion [15, 16, 22]. To increase the flexion gap, surgeons tend to increase the posterior tibial slope intra-operatively, using the anterior tibial cortex as a reference (anterior tibial cortex referencing, ACR) [22]. This technique lowers the TFJ line and affects the tension of the soft tissues. Based on clinical observations of the authors, a little increase in posterior tibial slope could increase the flexion gap considerably and cause laxity in flexion with subsequent aberrant kinematics. These factors may put the implant at increased risk of wear [24] and lead to (mid-)flexion instability. A different surgical approach is to plan the desired tibial slope pre-operatively, based on the knee system, and to reference the tibial bone cut from the midpoint of the tibial plateau (centre of tibial plateau referencing, CPR). In the latter case, the choice of the posterior tibial slope does not substantially alter the TFJ line at the centre of the cut surface of the proximal tibia [20]. Only a limited number of studies were found in the literature on the effect of posterior tibial slope in TKA, which show contradictory results [16, 19, 25, 26]. Also little attention was paid to the surgical referencing technique used, and the effect of tibial slope on the forces in the TFJ, PFJ and knee ligaments during activities of daily living (ADLs).

The purpose of this study was, therefore, to investigate the effect of alterations of the tibial slope on TFJ laxity and kinematics, quadriceps, patello-femoral and ligament forces during a demanding activity, and to elucidate possible differences arising from the surgical referencing technique. The hypothesis was that the tibial slope influences the knee laxity, kinematics and loads both in flexion and extension, and that the effect depends on the referencing technique. This study presents novel findings on the effects of tibial slope and surgical techniques on the biomechanics of the reconstructed knee, which may be translated directly into the clinical practice.

## Materials and methods

**Fig. 1:**
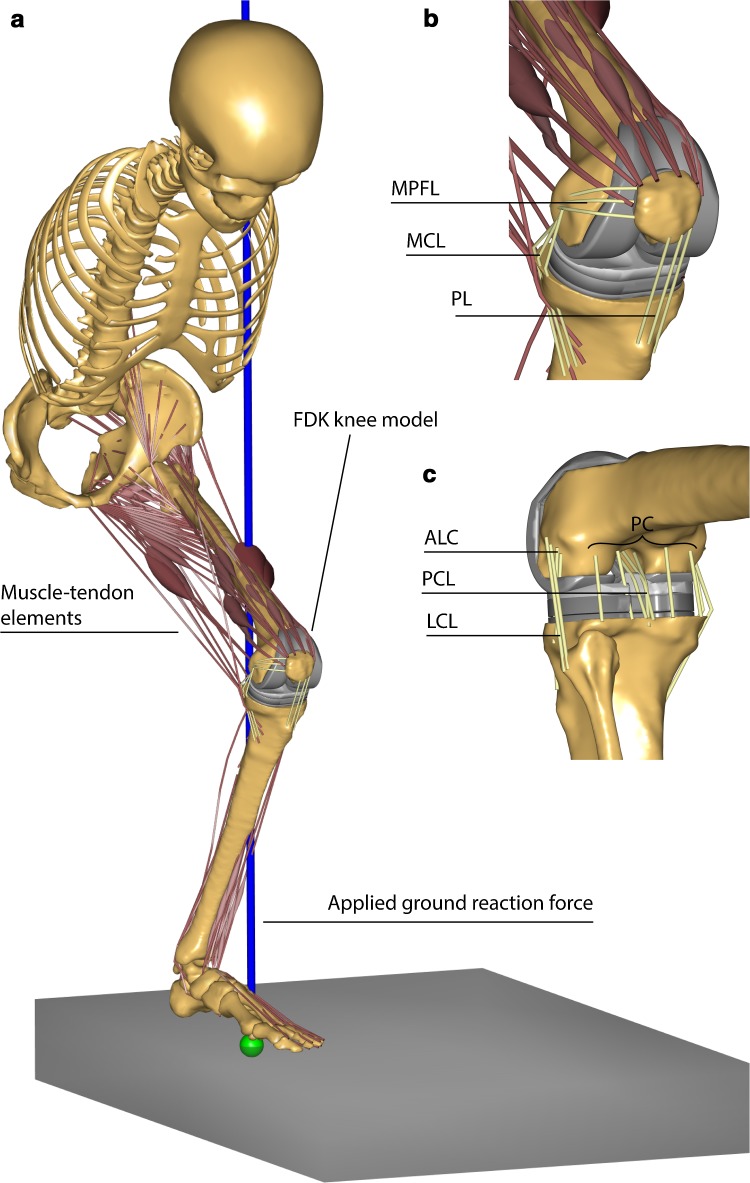
A snapshot of the musculoskeletal model used in this study. **a** The model includes head, trunk and the leg side with the implanted knee. The lower extremity is actuated by 166 muscle–tendon elements. Ground reaction forces and squatting kinematics are applied as input. The tibio- and patello-femoral joints are modelled using force-dependent kinematics (FDK) and include spring ligaments and articular surface contact: **b** antero-medial view of the knee with medial patello-femoral ligament (MPFL), medial collateral ligament (MCL) and patellar ligament (PL); **c** postero-lateral view of the knee with antero-lateral complex (ALC), posterior capsule (PC), lateral collateral ligament (LCL)

An existing patient-specific musculoskeletal model of CR-TKA was used for this study (Fig. 1) [17]. The model was previously validated against experimental measurements of TFJ contact forces during normal and right turn gait in a patient with a telemetric knee prosthesis, and sagittal plane kinematics during unloaded leg-swing trial under fluoroscopic examination [11]. Details of the model are provided in a separate Appendix. The tibial insert (Congruent NK-II CR, Zimmer Biomet, Warsaw, IN, USA) of the knee prosthesis had a conforming shape, with upwardly sloping lipped bearings at both the anterior and posterior rims of the insert. The post-operative posterior tibial slope was quantified as the angle of built-in slope of the tibial insert plus the angle of the post-operative tibial resection, measured relative to the tibial mechanical axis. The tibial mechanical axis was defined as the axis connecting the tibial intercondylar eminence, proximally, to the centre of the inferior tibial articular surface (tibial plafond), distally. The post-operative tibial slope was equal to 0° (neutral slope) and represented the baseline case for successive comparisons.

**Fig. 2:**
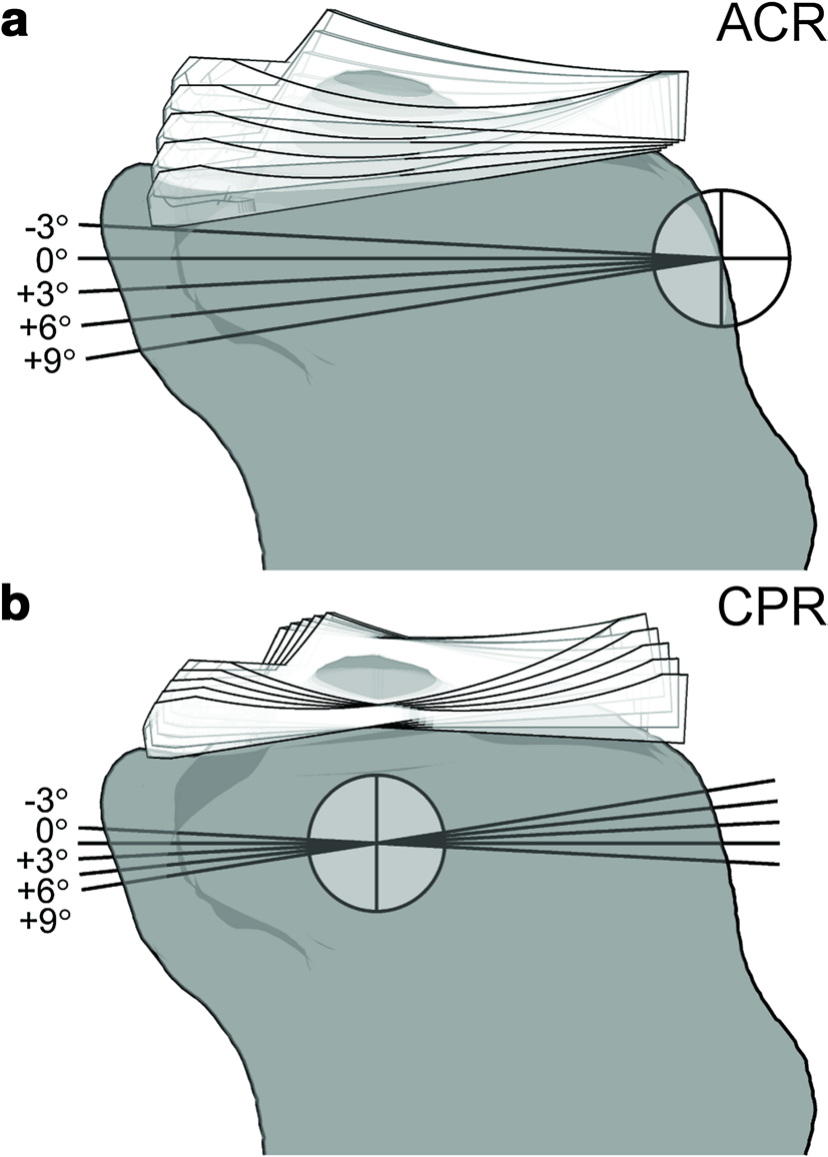
Schematic representation of **a** anterior tibial cortex referencing (ACR) and **b** centre of tibial plateau referencing (CPR) techniques used in this study to alter the tibial slope. The *crossed circles* represent the pivot point for the virtual resection. The outline of the tibial insert from −3° to +9° of tibial slope is superimposed. With ACR technique a more posterior tibial slope shifts all points on the tibial plateau distally, and a more anterior slope, proximally (**a**). With CPR technique, changing the degree of tibial slope does not alter the joint line in correspondence of the pivot point (**b**)

To assess the effect of surgical referencing technique, two series of analyses were designed. In the first series, the tibial slope was altered by rotating the tibial component on the sagittal plane around a pivot point located at the anterior aspect of the proximal tibia (ACR technique, Fig. 2a). With such technique, a more posterior tibial slope would shift distally all the points of the tibial plateau, and a more anterior slope, would shift them proximally. In the second series, the pivot point was defined as the midpoint between the centres of the medial and lateral tibial plateaus (CPR technique, Fig. 2b). In this situation, a more posterior tibial slope would shift distally all the points located posteriorly to the pivot point, and proximally all the points located anteriorly, and vice versa for a more anterior slope. In addition to the neutral slope case (0°), four more cases were analysed, three with more posterior slope (+3°, +6°, +9°) and one with more anterior slope (–3°), with both ACR and CPR technique, leading to five slope cases in each technique.

Anterior–posterior (AP) and varus–valgus (VV) laxity tests were simulated. An unloaded case was first analysed, in which the knee joint sought its own equilibrium position throughout a 0°–90° knee flexion range. The anterior tibial translation and varus angle were calculated according to Grood and Suntay’s joint coordinate system definition [13]. Subsequently, anterior and posterior forces of 70 N in magnitude [4] were applied to the proximal tibia, and the correspondent AP translations recorded. Similarly, varus and valgus loads of 15 N m in magnitude [14] were applied to the tibia by means of 50 N-forces, applied 30 cm below the TFJ line and directed medially and laterally, respectively. The correspondent varus and valgus rotations were recorded. Measurements of laxity were obtained at 0°, 30°, 60°, 90° of knee flexion. The anterior and posterior laxities were calculated as the tibial translation during the anterior and posterior laxity tests, respectively, minus the tibial translation in the unloaded case. The varus and valgus laxities were calculated as the knee angle during the varus and valgus laxity tests, respectively, minus the knee angle in the unloaded case.

To investigate the knee kinematics and forces under loading, a two-legged squatting motor task (PS_2legsquat1) from the fifth *Grand Challenge Competition To Predict In Vivo Knee Loads* dataset [11] was analysed, which started from a standing position, followed by a descending phase and an ascending phase. The range of knee flexion was approximately 0°–90°. Throughout the squatting task, the following outcomes were calculated: the displacement of the TFJ contact point, calculated as the centre of pressure of the medial and lateral TFJ contact forces, the forces in the MCL, LCL and PCL, the quadriceps muscle forces, the force exchanged between the quadriceps tendon and the femur condyles through wrapping (quadriceps–femur force), and the PFJ contact force.

## Results

### Laxity with ACR technique

**Fig. 3:**
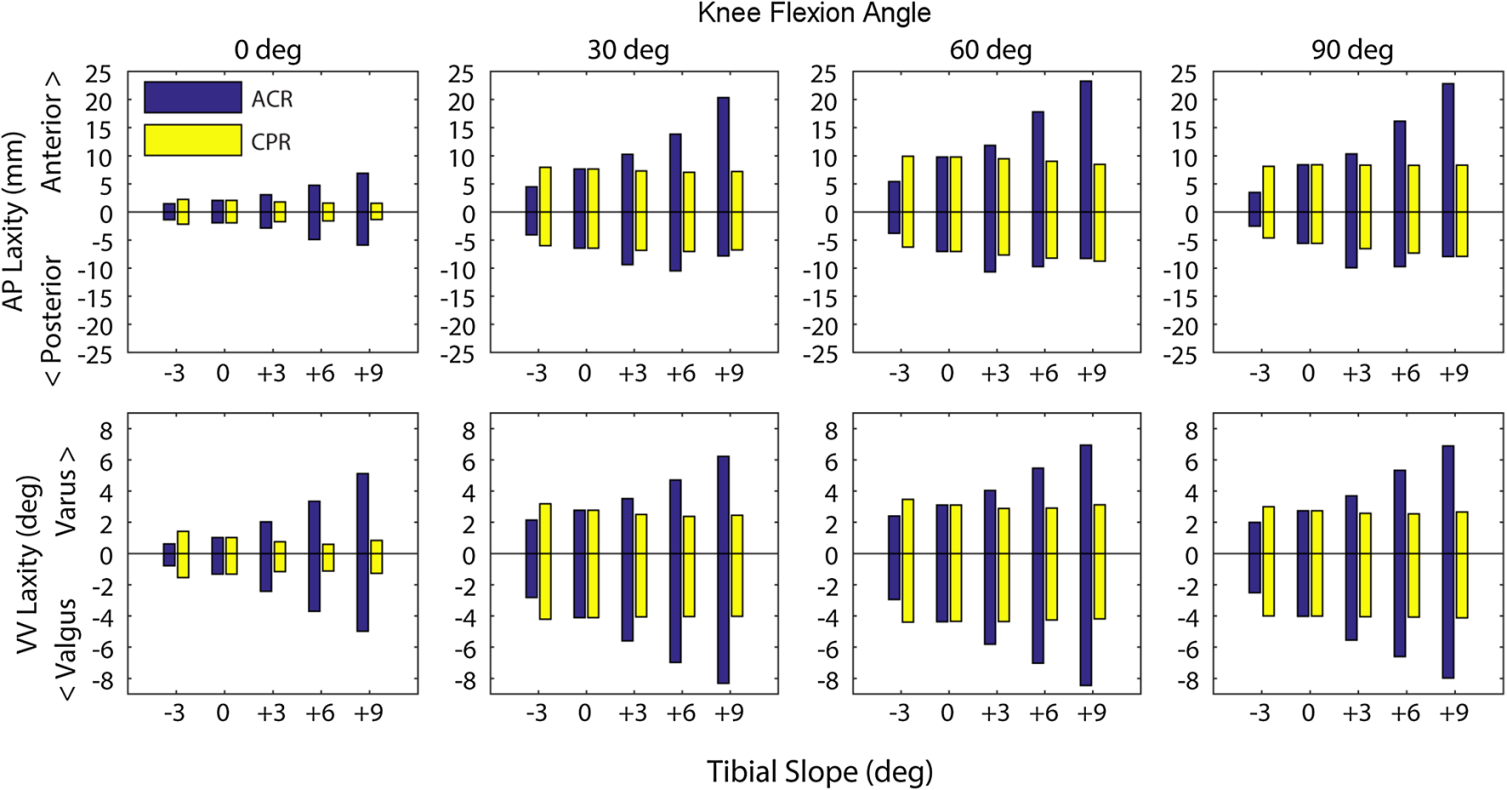
Anterior–posterior (*top*) and varus–valgus (*bottom*) laxities at 0°, 30°, 60° and 90° of knee flexion for different degrees of tibial slope and for anterior tibial cortex referencing (ACR) and centre of tibial plateau referencing (CPR) techniques. Note the large effect of tibial slope on laxity with ACR technique, and the minor effects of CPR technique

The anterior laxity increased with more posterior slope with the ACR technique, in all knee flexion angles (Fig. 3, blue series). The posterior laxity increased with more posterior slope, in extension, whereas it did not show a clear trend for the other flexion angles. Both valgus and varus laxities increased with more posterior slope in all knee flexion angles. Both anterior, posterior, valgus and varus laxities decreased with more anterior slope (−3°), in all knee flexion angles.

### Laxity with CPR technique

Knee laxity was less affected by changes in tibial slope with CPR technique (Fig. 3, yellow series), than with ACR technique. The anterior laxity decreased with more posterior tibial slope with the CPR technique, in all knee flexion angles. Valgus and varus laxities slightly decreased with more posterior slope, in extension. In all other knee flexion angles, only marginal changes (<0.4°) in valgus and varus laxity occurred with more posterior slope. A more anterior slope caused changes in laxity, which were less than 20% of the neutral case values, with the exception of the varus laxity in extension, which increased by 0.4° (+39%).

### Kinematics and loads during squatting

**Fig. 4:**
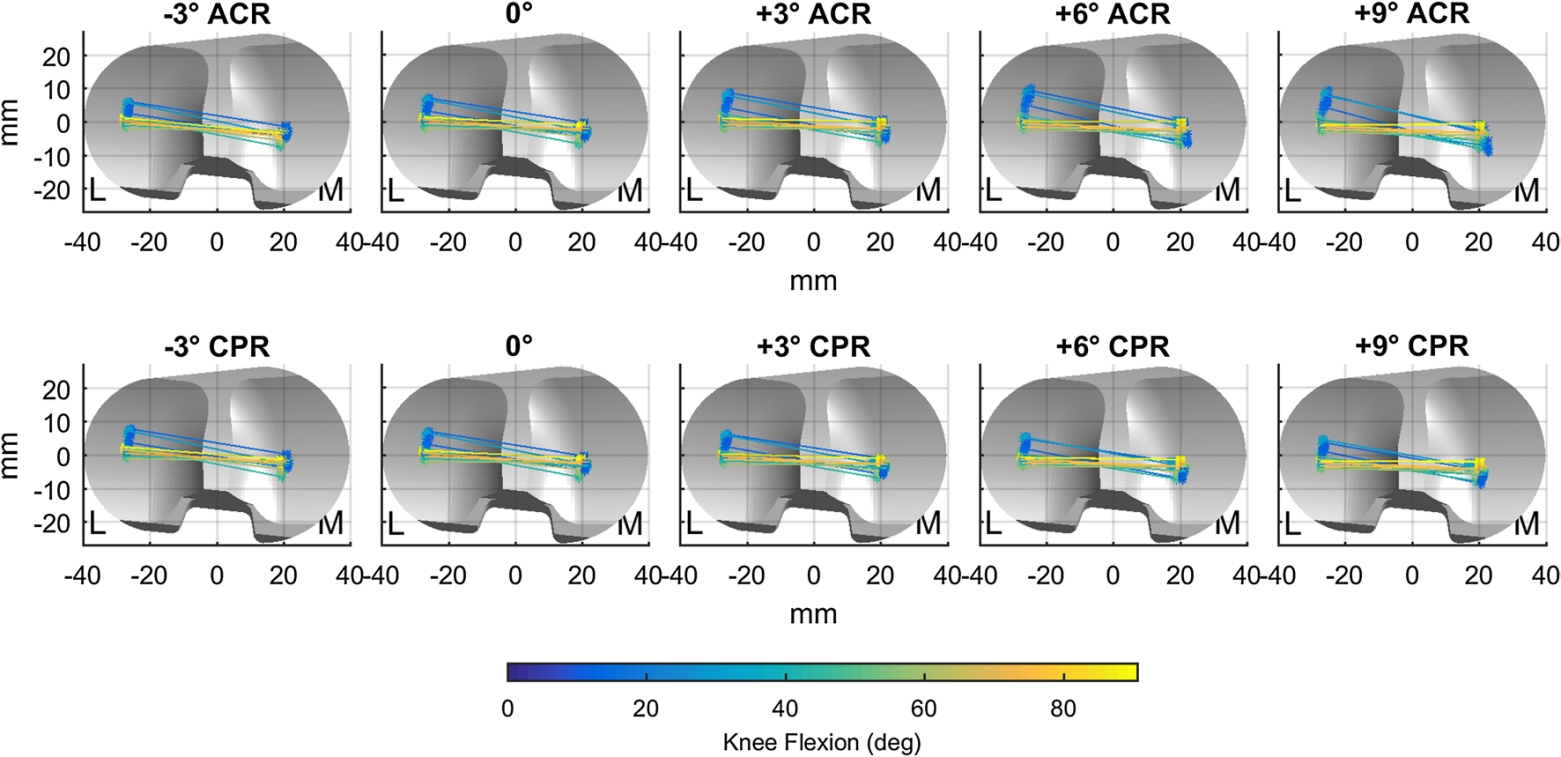
Displacement of the tibiofemoral contact point during squatting at baseline (0°), with an anterior slope (−3°) and posterior slope (+3°, +6°, +9°) and for anterior tibial cortex referencing (ACR, *top*) and centre of tibial plateau referencing (CPR, *bottom*) techniques. Contact points on the lateral (L) and medial (M) side of the tibial plateau are connected together and colour-coded according to the knee flexion angle

The distance travelled by the TFJ contact point increased on both medial and lateral side with more posterior tibial slope with ACR technique, and it was not altered substantially with the CPR technique (Fig. 4).

**Fig. 5:**
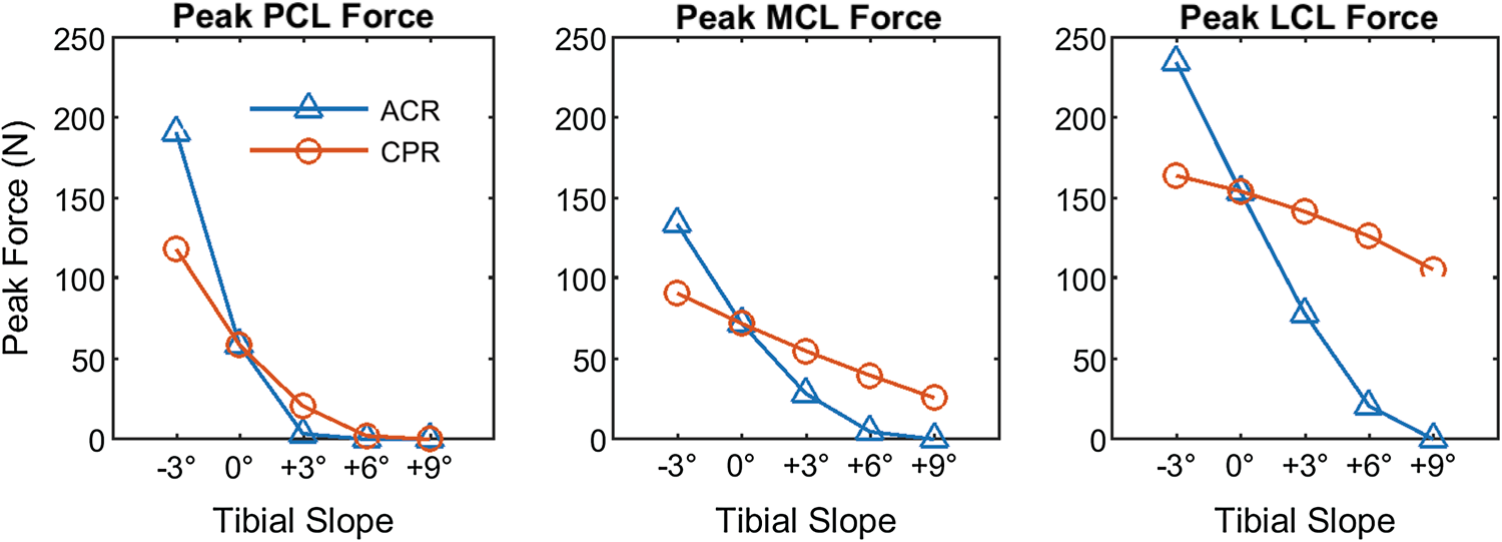
Peak posterior cruciate ligament (PCL, *left*), medial collateral ligament (MCL, middle) and lateral collateral ligament (LCL, *right*) force during squatting at varying degrees of tibial slope and referencing techniques. Note how the effect of tibial slope on knee ligaments is more moderate with CPR than with ACR technique

The peak PCL force during squatting decreased with more posterior slope with both referencing techniques (Fig. 5, left). Both medial and lateral collateral ligament peak forces decreased with more posterior slope (Fig. 5, centre and right). However, with the CPR technique neither ligament became slack, and the force reduction was more moderate, when compared to the ACR technique.

**Fig. 6:**
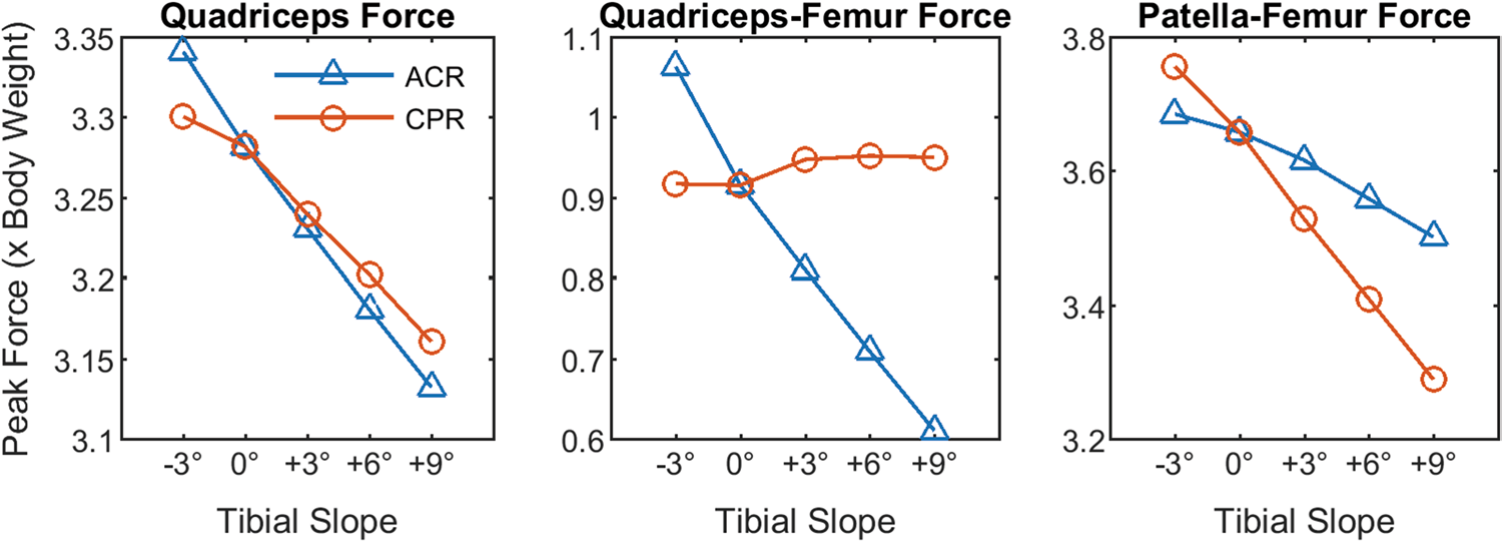
Effect of tibial slope on peak quadriceps (*left*), quadriceps–femur (*middle*) and patella–femur (*right*) forces during squatting. Note the different patterns of quadriceps–femur and patella–femur force between ACR and CPR techniques

The peak quadriceps force during squatting decreased by 5.2% body weight (BW), on average, for every 3° more posterior slope with the ACR technique (Fig. 6, left). Similarly, the quadriceps–femur force decreased by 11% BW (Fig. 6, centre), and the peak PFJ contact force decreased by 5% BW, on average (Fig. 6, right). With the CPR technique, the peak quadriceps force during squatting decreased by 3.5% BW, on average, for every 3° more posterior slope; the peak quadriceps–femur force slightly increased, and the peak PFJ contact force decreased by 12% BW, on average.

## Discussion

**Fig. 7:**
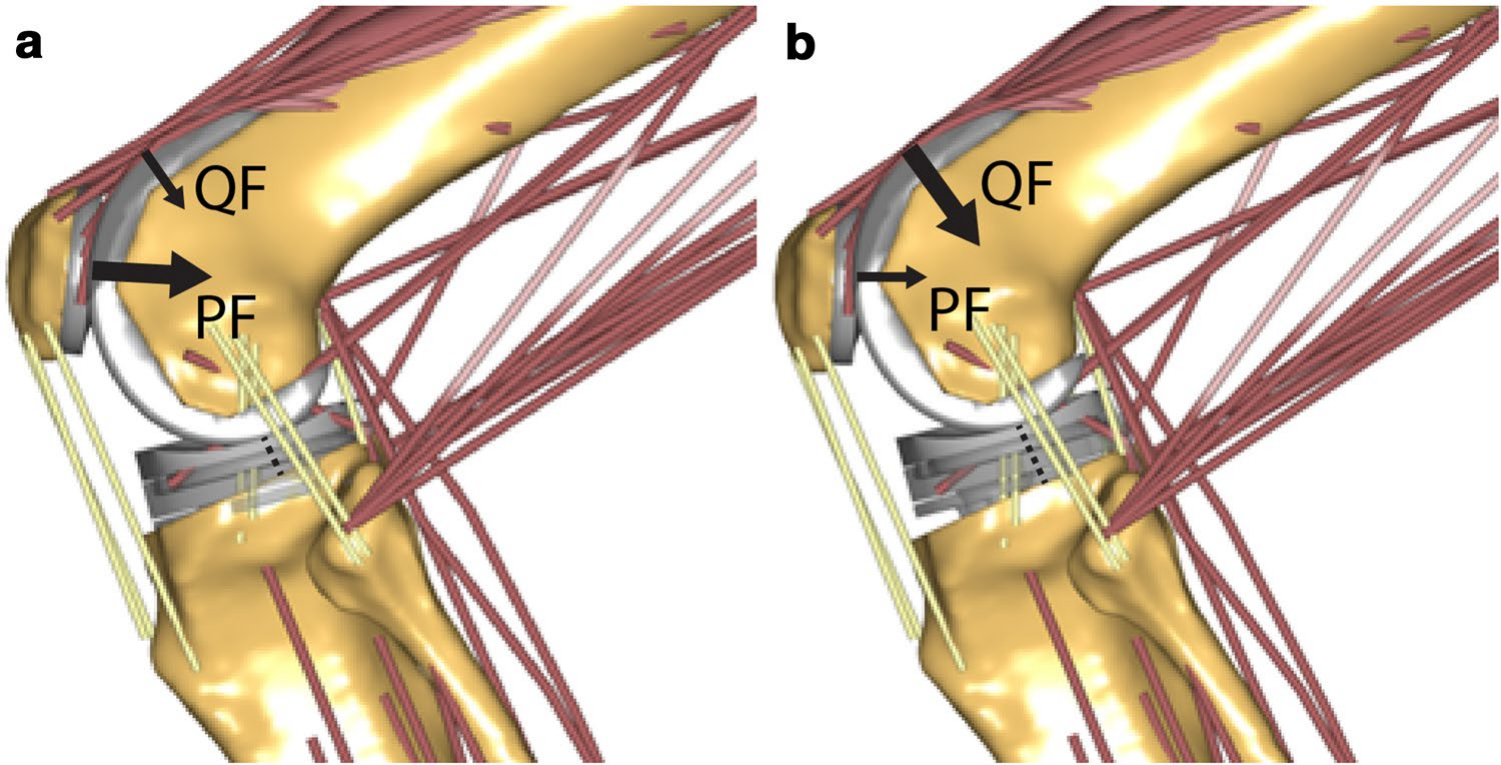
Conceptual representation of the quadriceps–femur (QF) and patella–femur (PFJ) load sharing with +9° of tibial slope with **a** anterior tibial cortex referencing (ACR) and **b** centre of tibial plateau referencing (CPR) techniques. The quadriceps muscle force decreases with more posterior slope both with ACR and CPR techniques. However, with ACR the position of the patella relative to the femur condyles is higher than with CPR, and a much lower quadriceps force can be transmitted via the quadriceps tendon directly through the femur, thus the patella–femur force is reduced only by a little amount; with CPR technique the amount of quadriceps force transmitted through the femur is higher, thus the patella–femur force is reduced more importantly

The main finding of this study was that the knee laxity increased substantially, both in flexion and, unexpectedly, also in extension with more posterior tibial slope in case the ACR technique was simulated. In contrast, varying the tibial slope with the CPR technique had little effects on knee laxity. The tension of the TFJ ligaments during simulated squat decreased with more posterior tibial slope for both referencing techniques, indicating a progressive loosening of the TFJ gap with more posterior slope, although the changes were more distinct with the ACR technique. Also a previous study found decreased PCL strain with more posterior tibial slope [22]. No aberrant TFJ contact point movements were observed during simulated squat, even with large degrees of posterior tibial slope, when the CPR technique was simulated. This is in agreement with a previous fluoroscopic study on knee kinematics during stair ascent, getting up from and sitting down on a chair and single-leg climbing up a step [6], in which the original slope was restored and the post-operative slope ranged from −2° to 10°. Therefore, the concern for increased risk of wear, due to extreme roll-back with more posterior tibial slope [24], does not appear much justified, based on results of this study, as long as the TFJ gap is successfully balanced in both flexion and extension. In contrast, more slope with the ACR technique resulted in larger excursions of the TFJ contact point in extension on both medial and lateral side, owing to a loosened TFJ gap, which in turn may lead to increased wear of the polyethylene insert. A more posterior location of the TFJ contact point can, in principle, increase the quadriceps moment arm and reduce the quadriceps force [7, 10]. Previous studies found more posterior TFJ contact point (in vivo) [12] and reduced quadriceps forces (ex vivo) [21] with more posterior tibial slope. In the present study, the peak quadriceps forces were reduced during squatting with more posterior tibial slope with both referencing techniques. The patella shifted superiorly relative to the femur condyles with more posterior tibial slope with the ACR technique, due to lowering of the TFJ line. This reduced the force exchanged between quadriceps tendon and femoral condyles by wrapping (Fig. 7, centre). With the CPR technique, the patellar height remained unchanged, and the quadriceps–femur load sharing was preserved. Also the PFJ contact force was more effectively reduced with more posterior tibial slope with the CPR technique (−12% BW every 3°) relative to the ACR technique (−5% BW every 3°). Although the difference was relatively small, decreased PFJ contact forces may contribute to reduce anterior knee pain and implant wear [3] after following CR-TKA.

In the present study, a validated patient-specific musculoskeletal model was used to simulate laxity tests and a squatting activity, under different tibial slope conditions. Furthermore, a highly controlled and parameterised study design was adopted, in which the degree of tibial slope and the referencing technique were the sole variables. A major strength of this approach lies in the possibility to isolate the effect of tibial slope from the effect of all the other variables that may possibly affect the outcomes of TKA, and which in previous literature may have acted as confounders. Distinct biomechanical pathways could be identified from altering the tibial slope with different referencing techniques. Also previous studies found important effects of tibial slope on knee laxity, especially those which used a referencing technique similar to the ACR of this study [16, 22]. Conversely, more posterior tibial slope obtained with the CPR technique did not cause such big effects on TFJ laxity, in the present study. Also Whiteside and Amador found no major differences in the mean knee laxity parameters after increasing the posterior tibial slope [25], using posteriorly sloped tibial inserts. Their method did not alter the level of the TFJ line in correspondence of the centre of the tibial plateaus and is analogous to the CPR technique simulated in this study.

Posterior tibial slope has been proposed as a viable alternative to PCL release to address flexion gap tightness in CR-TKA [16, 26]. In the present study, it was shown that a more posterior slope with CPR technique loosens the TFJ in flexion. This finding is supported by the study of Okazaki et al. [20], which shows an increase in the flexion gap of 2 mm for every 5° increase in the posterior tibial slope, using the CPR technique. In any case, the increase in tibial slope should never be at the expenses of the tibial insertions of PCL [18]. In CR-TKA, the PCL has a major role in governing the stability of the TFJ [23]. Therefore, caution should be used when performing the proximal tibial resection with an increased slope, and the appropriate surgical tools should be used to prevent damage of the PCL tibial insertion.

Interestingly a more posterior tibial slope, obtained with the ACR technique—which is meant to address a flexion tightness problem—produces, in fact, remarkable effects also in extension. This can be explained by noting that the ACR technique shifts all points of the tibial plateau distally, which, in turn, loosens the TFJ throughout the whole knee flexion–extension range. In clinical practice, if the extension gap has been already successfully balanced, any further increase in the posterior tibial slope (using the ACR technique) will alter the level of the TFJ line, which reduces the tension of the soft tissues (MCL, LCL and PCL), and increases the laxity of the knee both in flexion and in extension. Surgeons should, therefore, pay much attention when altering the tibial slope using the ACR technique, as it may have serious consequences for the overall stability of the knee.

The results of this study suggest that a thorough pre-planning of the desired tibial slope should be made, which considers both the type of implant available and the surgical technique utilised, and that the execution of the tibial resections should be as accurate as possible. One factor in the decision is the knee system used. Some systems have tibial inserts available with a built-in slope of 3°–4°. With these systems, the surgeon should be careful to aim for too much slope with the primary cut, and rather aim for 0°–3°. With other systems, that do not have slope in the insert, the surgeon could aim for more slope, e.g. 3°–6° in the tibial cut, but referenced from the centre of the tibial plateau. Another factor is the native tibial slope of the patient. When a patient has a large native tibial slope, e.g. 10°, the surgeon can anticipate a tight knee in flexion when using a limited sloped cut. A pre-operatively planned slope, referenced from the centre of the tibial plateau, may help creating a correct balance and prevent the disadvantages of the anterior referenced slope for such a patient. The native tibial slope of the present case study was about 7°, as measured on the available pre-operative CT images. Interestingly, the simulated cases with +6° and +9° of slope with CPR technique—being the nearest to the native slope—both provided the best biomechanical results. The native tibial slope may be an important parameter to take into account in the pre-operative planning, which is also very easy to measure on pre-operative radiographs.

Obviously, this study had several limitations. The musculoskeletal computer model represented a CR-TKA, and the results should not be generalised to other implant designs such as the PS-TKA. Although the principles behind knee surgery are similar, the mechanisms by which stability is achieved in flexion are radically different, as the CR-TKA relies on the preservation of the PCL, whereas the PS-TKA relies on a post-cam mechanism embedded in the prosthesis.

Nearly complete slackening of the PCL was observed in the preset study, already with +3° slope. This effect may be due to the strain parameters assigned to the bundles of the PCL. It should be noted that the sagittal plane knee kinematics were validated against experimental measurements of a free leg-swing fluoroscopy trial, which showed some signs of PCL laxity. It is plausible that the parameters of the PCL of the present model reflected those of a relatively lax PCL, and that knees with a perfectly functional PCL after CR-TKA will likely exhibit less slackening of the PCL in flexion.

The effects of tibial slope on the knee biomechanics were analysed during a single squatting motor task, by simulating multiple cases of tibial slope. In principle, it would be possible to analyse also other motor tasks, e.g. corresponding to several ADLs, but this would require additional computer analyses, which would add to the computation time. A squatting motion was chosen for the simulations, since it included both a wide range of flexion–extension and a significant muscular endeavour around the knee joint. Care should also be taken not to extrapolate the results of the present study to deep flexion, as this range was not investigated.

When analysing squatting, it was assumed that the overall body kinematics and loading conditions would hold among different configurations of the tibial slope. Some neuro-motor adaptations may occur as a result of alterations of the prosthesis alignment, but the study of those fell out of the scope of the present study. Nevertheless, the computational analysis technique employed allowed to predict changes in knee kinematics and forces, resulting from changes in the tibial slope, as intended.

## Conclusion

Changes in posterior tibial slope have considerable effects on knee laxity, kinematics and forces. More posterior tibial slope with the ACR technique increases the knee laxity in flexion, but also, unexpectedly, in extension. More posterior slope with CPR technique leaves the knee laxity almost uninfluenced, is beneficial for the knee extensor apparatus and relieves the pressure on the PFJ. The tibial resection should be pre-planned and executed as accurately as possible using CPR technique.

## Electronic supplementary material

Below is the link to the electronic supplementary material.

Supplementary material 1 (DOCX 39 kb)

Supplementary material 2 (WMV 16814 kb)

Supplementary material 3 (WMV 33853 kb)
