# Supplementary material for: Anterior referencing of tibial slope in total knee arthroplasty considerably influences knee kinematics: a musculoskeletal simulation study
Source: Knee Surg Sports Traumatol Arthrosc. 2017 May 12;26(5):1540–8. doi: 10.1007/s00167-017-4561-3 (PMC5907627; doi:10.1007/s00167-017-4561-3)
Supplement: Supplementary file 1 — Supplementary material 1 (DOCX 39 kb) [file 167_2017_4561_MOESM1_ESM.docx]

**APPENDIX: Detailed musculoskeletal model description**

The model of CR-TKA used in this study was built upon the Twente Lower Extremity Model 2.0 (TLEM 2.0) template for subject-specific models [4], which included head, trunk, two arms and two legs, connected by idealised joints (Fig. 1). The lower extremities were scaled to the patient’s morphology, using patient-specific medical images. Three-dimensional models of the CR-TKA implant were incorporated into the patient’s left knee. Reflective skin marker trajectories from the available motion capture data were used as input to derive the kinematics of the idealised joint degrees-of-freedom (DOFs), using a motion optimization algorithm [1]. Subsequently, the TFJ and PFJ constraints were removed, and ligaments and articular surface contacts introduced to provide stiffness and support to the unconstrained joints. Recorded ground reaction forces and moments (GRF&M) were input to an inverse-dynamic model, actuated by 166 Hill-type muscle-tendon elements. Muscle forces, ligament forces and articular contact forces were solved simultaneously using inverse-dynamic coupled with force-dependent kinematic (FDK) analyses [2]. Some variations were introduced with respect to a previously published model [6]: the patellar ligament (PL) was modelled as three non-linear elastic springs with large stiffness, in place of a rigid rod; the lateral PFJ ligament bundles were removed from the analysis to save computation time, since they were found to remain slack throughout a series of trial model analyses; the path of the muscle *vastus medialis* was further optimised by means of an ellipsoidal wrapping object, to account for the obliquity of the fascicles at its patellar insertion [3, 5].

**REFERENCES**

1. Andersen MS, Damsgaard M, Rasmussen J (2009) Kinematic analysis of over-determinate biomechanical systems. Comput Methods Biomech Biomed Engin 12:371–384

2. Andersen MS, Damsgaard M, Rasmussen J (2011) Force-dependent kinematics: a new analysis method for non-conforming joints. XIII Int Symp Comput Simul Biomech Leuven, Belgium

3. Bennett WF, Doherty N, Hallisey MJ, Fulkerson JP (1993) Insertion orientation of terminal vastus lateralis obliquus and vastus medialis obliquus muscle fibers in human knees. Clin Anat 6:129–134

4. Carbone V, Fluit R, Pellikaan P, van der Krogt MM, Janssen D, Damsgaard M, Vigneron L, Feilkas T, Koopman HFJM, Verdonschot N (2015) TLEM 2.0 – A comprehensive musculoskeletal geometry dataset for subject-specific modeling of lower extremity. J Biomech Elsevier 48:734–741

5. Engelina S, Antonios T, Robertson CJ, Killingback A, Adds PJ (2014) Ultrasound investigation of vastus medialis oblique muscle architecture: An in vivo study. Clin Anat 27:1076–1084

6. Marra MA, Vanheule V, Fluit R, Koopman BHFJM, Rasmussen J, Verdonschot N, Andersen MS (2015) A subject-specific musculoskeletal modeling framework to predict in vivo mechanics of total knee arthroplasty. J Biomech Eng 137:20904
